# A baseline waterbird occurrence dataset for Miyun Reservoir, Beijing, China

**DOI:** 10.3897/BDJ.14.e203213

**Published:** 2026-08-17

**Authors:** Weiwei Sun, Qun Wang, Chenqiang Zhao, Lei Liu, Dehai Gu, Zheping Xu, Fanjuan Meng, Lei Cao

**Affiliations:** 1 Beijing Miyun Reservoir Administration Management Office, Beijing, China Beijing Miyun Reservoir Administration Management Office Beijing China; 2 State Key Laboratory of Regional and Urban Ecology, Research Centre for Eco-Environmental Sciences, Chinese Academy of Sciences, Beijing, China State Key Laboratory of Regional and Urban Ecology, Research Centre for Eco-Environmental Sciences, Chinese Academy of Sciences Beijing China; 3 National Science Library, Chinese Academy of Sciences, Beijing, China National Science Library, Chinese Academy of Sciences Beijing China; 4 University of Chinese Academy of Sciences, Beijing, China University of Chinese Academy of Sciences Beijing China

**Keywords:** annual life cycle, GBIF, sampling-event dataset, synchronous waterbird census

## Abstract

**Background:**

Miyun Reservoir is north China’s largest artificial reservoir and vital drinking water supply for Beijing and is a key stopover for migratory birds along the East Asian-Australasian Flyway (EAAF). Previous literature indicates that continuous high-water-level regulation starting in 2015 may alter shallow wetland habitats and migratory bird distribution patterns; previous studies reported a decline in reservoir waterbird occupancy from 79% (2018) to 56% (2022). However, no complete annual-cycle synchronous waterbird census covering the full reservoir basin has been published to date. This paper releases standardised synchronous survey data collected across Miyun Reservoir in 2023. This one-year dataset provides a spatial and temporal baseline for future multi-year ecological modelling and comparative analysis, rather than direct evidence to quantify long-term ecological changes driven by reservoir water management.

**New information:**

We publish the first full annual-cycle standardised waterbird dataset covering all 14 fixed sampling stations of Miyun Reservoir, with four seasonal synchronous surveys implemented between 10 April and 16 October 2023. A total of 32 waterbird species and 25,180 individuals were recorded, generating 198 unique Darwin Core-compliant occurrence records archived on GBIF with a permanent DOI (https://doi.org/10.15468/vfjx35). The dataset delivers baseline abundance and distribution data for resident and migratory waterbird assemblages within the reservoir basin.

## Introduction

Miyun Reservoir is the largest reservoir in north China and the primary drinking water source for Beijing, approximately 13 km north of Miyun District at the junction of the Chao and Bai Rivers (Zhou 2024). The total water surface area reaches around 188 km² with a 200 km shoreline perimeter (Zhang et al. 2023).

Waterbird communities serve robust bioindicators of wetland ecosystem health via species richness and population metrics (Gregory and Strien 2020). Situated on the East Asian-Australasian Flyway (EAAF), the reservoir functions as a critical migratory stopover and regional biodiversity hotspot (Liang et al. 2022). The vulnerable White-naped Crane *(Antigone vipio*) was documented with 1020 individuals (Lei 2013) in a 2013 regional wetland survey, demonstrating the site’s international wetland conservation value (Mundkur and Nagy 2012).

Since September 2015, the Middle Route of the South-to-North Water Diversion Project began supplying water to Miyun Reservoir. Combined with upstream flood inflow in 2021, the reservoir water level rose from ~ 135 m to a record 155.3 m Dagu elevation and has been maintained at this high level as a standardised long-term water storage regulation regime (Zhang et al. 2023, Liu et al. 2024, Zhou 2024). Previous studies have reported that sustained high water levels may have submerged extensive near-shore farmland, tidal flats and riparian shrub wetlands, potentially reducing shallow-water foraging habitats suited to many waterbird taxa. Published satellite tracking research indicated decreasing migratory bird site visitation rates, alongside shifts in phytoplankton, macrophyte assemblages and reservoir water nitrogen concentrations (Zhao et al. 2022, Yi et al. 2023, Xue et al. 2024). Earlier bird surveys (2020–2022) recorded 324 total avian species across the reservoir and peripheral tributaries, but lacked reservoir-wide synchronous waterbird sampling covering full seasonal migration cycles (Li et al. 2022, Han et al. 2025). The absence of unified, basin-wide annual waterbird baseline data limits the development of ecological response frameworks for subsequent long-term water level management assessments (Zhou et al. 2020). Standardised synchronous census protocols can reduce analytical bias and support cross-study comparison (Thomas 1996, Thomas and Martin 1996, Atkinson et al. 2006, Gosbell and Clemens 2006). This study helps fill the geographic gap of prior partial northern-reservoir surveys and releases complete 2023 seasonal waterbird observation data for the entire reservoir basin.

**Clarification of dataset positioning**

This manuscript exclusively provides 2023 one-year observational waterbird data. The dataset cannot independently establish causal relationships between long-term high-water regulation and interannual waterbird community variation. Its core function is to act as standardised baseline material for follow-up multi-year monitoring and ecological modelling, rather than a quantitative impact evaluation of reservoir water management policies.

The complete absence of White-naped Cranes during the four seasonal synchronous reservoir-wide surveys conducted in 2023 contrasts with the historical record of 1,020 individuals documented at Miyun Reservoir in 2013. Whether this reflects interannual population fluctuations, habitat changes potentially associated with prolonged high-water-level regulation or other ecological factors cannot be determined from this one-year baseline dataset. Continued standardised annual monitoring will be necessary to evaluate future population trends of this vulnerable species.

## General description

### Purpose

Publicly available Darwin Core sampling-event occurrence dataset generated from four synchronous seasonal waterbird surveys across Miyun Reservoir in 2023.

## Sampling methods

### Study extent

The area surveyed consists of Miyun Reservoir, Beijing, China. A total of 14 sites were surveyed to cover the whole area.

### Sampling description

Surveyed waterbird taxa followed the taxonomy adopted in *Waterbird Population Estimates* (*5^th^ Edition*) (Mundkur and Nagy 2012), the Ramsar Convention’s reference for identifying internationally important wetland bird concentrations. Four seasonal synchronous surveys were conducted at 14 fixed permanent stations covering the full reservoir basin.

### Quality control

Large mixed bird flocks commonly cause visual counting underestimation (Rappoldt et al. 1985). No exceptionally large aggregations were encountered during fieldwork. To minimise missing individuals, the reservoir was divided into seven non-overlapping survey zones with comprehensive dyke and gully vantage point coverage. All censuses were completed by two trained professional observers equipped with high-magnification optical gear. While inter/intra-observer duplicate parallel counts were not performed, standardised fixed patrol routes and multi-angle observation minimised the undercount risk. Minor missing records may exist in remote, hard-to-access gully shorelines despite full coverage efforts.

### Step description

All field teams used Swarovski EL10×32SV binoculars and ATS80 telescopes (20–60× zoom). Survey protocols matched standardised workflows documented for the Huai River floodplain waterbird dataset (Wijethunge et al. 2025). Station selection adopted a three-stage screening process: 1) Satellite remote sensing mapping to delineate wetland zones and generate GPS navigation coordinates; 2) Local forestry bureau and wildlife expert consultation to identify high-abundance bird hotspots; and 3) Prioritisation of road-accessible shorelines, with foot surveys for remote areas. Observers positioned themselves with sunlight behind their backs and downwind under favourable meteorological conditions. Standard station spacing was set at 3–4 km (matching the 2–3 km reliable identification distance), with reduced intervals during fog or heavy haze. The reservoir was split into seven independent zones with continuous visual scanning between fixed points to eliminate observation gaps between stations. Daily survey window: 07:00–17:30 h. Each sampling station received one standardised survey per season, with a fixed observation duration of 20–30 minutes per site. Total surveyed area: 188,000,000 m².

**Exact seasonal survey dates**

1) Spring: 10–11 April 2023; 2) Summer: 24–25 July 2023; 3) Autumn: 04 September 2023; 4) Late autumn / early winter: 16 October 2023. Mid-October falls within the north China migratory window; many waterbird species have not yet settled into stable wintering habitats, so this survey phase likely underestimates resident winter waterbird abundance.

## Geographic coverage

### Description

**Figure 1.**
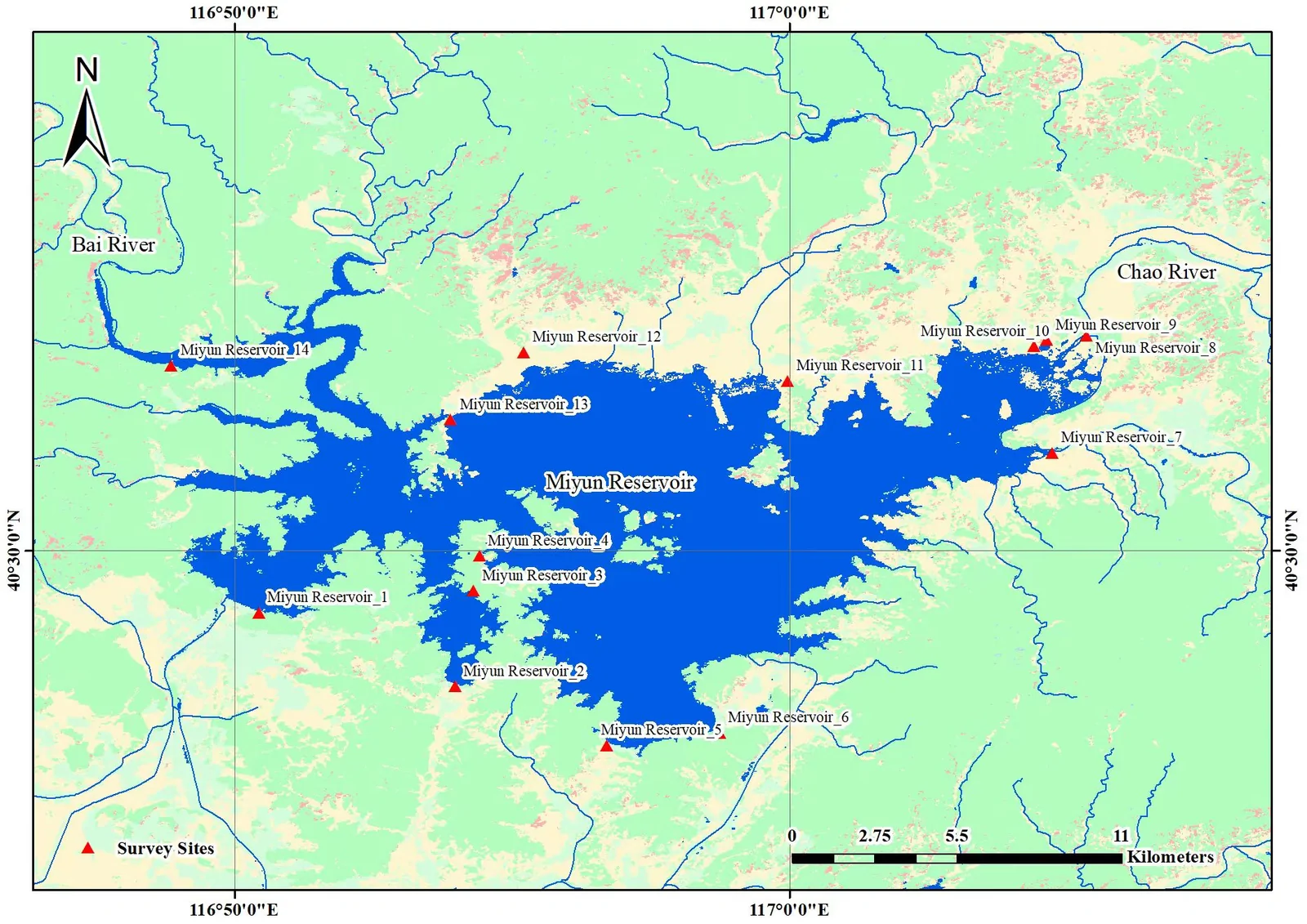
Spatial distribution of 14 fixed waterbird sampling sites across Miyun Reservoir. The map contains reservoir boundary, Bai and Chao inflow rivers, labelled station IDs matching dataset eventID, scale bar and north arrow.

The Miyun Reservoir, China (Fig. 1). Our seasonal surveys were implemented across all 14 fixed sampling locations. The coordinate range 40.442–40.564° N, 116.814–117.089° E corresponds to the central centroid coordinates of the 14 individual sampling sites, not the overall geographic bounding box of the reservoir. Fig. 1 displays all labelled survey locations consistent with dataset eventID, complete reservoir outline, Bai/Chao inflow rivers, scale bar and north arrow.

### Coordinates

40.442 and 40.564 Latitude; 116.814 and 117.089 Longitude.

## Taxonomic coverage

### Description

Four seasonal surveys in 2023 recorded a total of 32 valid waterbird species. Seasonal population statistics: Spring (mid-April): 20 species, 9,134 individuals; Summer (late July): 15 species, 8,998 individuals; Autumn (early September): 14 species, 1,232 individuals; Late autumn / early winter (mid-October): 14 species, 5,816 individuals.

No individuals of the White-naped Crane were recorded across the four seasonal synchronous surveys conducted in 2023, despite a historical census documenting 1,020 individuals at Miyun Reservoir in 2013 (Lei 2013).

### Taxa included

**Table:** 

Rank	Scientific Name	
species	*Actitis hypoleucos* Linnaeus, 1758	
species	*Aix galericulata* (Linnaeus, 1758)	
species	*Anas crecca* Linnaeus, 1758	
species	*Anas platyrhynchos* Linnaeus, 1758	
species	*Anas zonorhyncha* Swinhoe, 1866	
species	*Anser fabalis* (Latham, 1787)	
species	*Ardea alba* Linnaeus, 1758	
species	*Ardea cinerea* Linnaeus, 1758	
species	*Ardeola bacchus* (Bonaparte, 1855)	
species	*Chlidonias hybrida* Pallas, 1771	
species	*Chroicocephalus ridibundus* Linnaeus, 1766	
species	*Cygnus cygnus* Linnaeus, 1758	
species	*Egretta garzetta* (Linnaeus, 1766)	
species	*Fulica atra* Linnaeus, 1758	
species	*Larus argentatus* Pontoppidan, 1763	
species	*Mareca falcata* Georgi, 1775	
species	*Mareca strepera* Linnaeus, 1758	
species	*Mergellus albellus* (Linnaeus, 1758)	
species	*Mergus merganser* Linnaeus, 1758	
species	*Nycticorax nycticorax* (Linnaeus, 1758)	
species	*Phalacrocorax carbo* (Linnaeus, 1758)	
species	*Platalea leucorodia* Linnaeus, 1758	
species	*Podiceps cristatus* (Linnaeus, 1758)	
species	*Podiceps nigricollis* (Brehm, 1831)	
species	*Spatula clypeata* Linnaeus, 1758	
species	*Sterna hirundo* Linnaeus, 1758	
species	*Tachybaptus ruficollis* (Pallas, 1764)	
species	*Tadorna ferruginea* (Pallas, 1764)	
species	*Tadorna tadorna* Linnaeus, 1758	
species	*Tringa ochropus* Linnaeus, 1758	
species	*Vanellus cinereus* (Blyth, 1842)	
species	*Vanellus vanellus* (Linnaeus, 1758)	

## Temporal coverage

**Data range:** 2023-4-10 – 2023-10-16.

## Usage licence

### Usage licence

Other

### IP rights notes

Creative Commons Attribution Non-Commercial (CC-BY-NC) 4.0 License

## Data resources

### Data package title

Occurrence dataset from the waterbird survey of Miyun Reservoir, Beijing, China

### Resource link

https://www.gbif.org/dataset/af2ab816-37b6-4fa6-989b-7448bc14599c

### Number of data sets

1

### Data set 1.

#### Data set name

Occurrence dataset from the waterbird survey of Miyun Reservoir, Beijing, China

#### Data format

Darwin Core Sampling Event

#### Download URL

https://www.gbifchina.org.cn/archive.do?r=occurrence_dataset_from_the_2023_waterbird_survey_in_miyun_reservoir&v=1.6

#### Description

This dataset originates from the waterbird survey of Miyun Reservoir, Beijing, China, in 2023 (Sun et al. 2026). It contains detailed species, abundance and geographical information of the four seasons used by waterbirds as breeding grounds, wintering grounds and staging sites during the spring and autumn migration of their annual life cycle.

**Data set 1.** 

Column label	Column description
eventID (Event Core, Occurrence Extension)	An identifier for the broader dwc:Event that groups this and potentially other dwc:Events.
parentEventID (Event Core)	An identifier for the broader dwc:Event that groups this and potentially other dwc:Events.
samplingProtocol (Event Core)	The names of, references to or descriptions of the methods or protocols used during a dwc:Event.
sampleSizeValue (Event Core)	A numeric value for a measurement of the size (time duration, length, area or volume) of a sample in a sampling dwc:Event.
sampleSizeUnit (Event Core)	The unit of measurement of the size (time duration, length, area or volume) of a sample in a sampling dwc:Event.
samplingEffort (Event Core)	The amount of effort expended during a dwc:Event.
eventDate (Event Core)	The date-time or interval during which a dwc:Event occurred.
continent (Event Core)	The name of the continent in which the dcterms:Location occurs.
country (Event Core)	The name of the country or major administrative unit in which the dcterms:Location occurs.
countryCode (Event Core)	The standard code for the country in which the dcterms:Location occurs.
stateProvince (Event Core)	The name of the next smaller administrative region than country (state, province, canton, department, region etc.) in which the dcterms:Location occurs.
county (Event Core)	The full, unabbreviated name of the next smaller administrative region than stateProvince (county, shire, department etc.) in which the dcterms:Location occurs.
locality (Event Core)	The specific description of the place.
decimalLatitude (Event Core)	The geographic longitude (in decimal degrees, using the spatial reference system given in dwc:geodeticDatum) of the geographic centre of a dcterms:Location.
decimalLongitude (Event Core)	The geographic longitude (in decimal degrees, using the spatial reference system given in dwc:geodeticDatum) of the geographic centre of a dcterms:Location.
geodeticDatum (Event Core)	The ellipsoid, geodetic datum or spatial reference system (SRS) upon which the geographic coordinates given in dwc:decimalLatitude and dwc:decimalLongitude are based.
coordinateUncertaintyInMetres (Event Core)	The horizontal distance (in metres) from the given dwc:decimalLatitude and dwc:decimalLongitude describing the smallest circle containing the whole of the dcterms:Location.
locationID (Event Core)	An identifier for the set of dcterms:Location information. A unique identifier specific to this dataset, used to distinguish each fixed waterbird survey observation point.
basisOfRecord (Occurrence Extension)	The specific nature of the data record.
dynamicProperties (Occurrence Extension)	A list of additional measurements, facts, characteristics or assertions about the record. Meant to provide a mechanism for structured content. In this waterbird survey dataset, this field is used to record the IUCN Red List conservation category of each observed waterbird species in structured JSON format.
occurrenceID (Occurrence Extension)	An identifier for the dwc: Occurrence (as opposed to a particular digital record of the dwc: Occurrence).
individualCount (Occurrence Extension)	The number of individuals present at the time of the dwc: Occurrence.
organismQuantity (Occurrence Extension)	A number or enumeration value for the quantity of dwc: Organisms.
organismQuantityType (Occurrence Extension)	The type of quantification system used for the quantity of dwc: Organisms.
occurrenceStatus (Occurrence Extension)	A statement about the presence or absence of a dwc:Taxon at a dcterms: Location.
scientificName (Occurrence Extension)	The full scientific name, with authorship and date information if known.
kingdom (Occurrence Extension)	The full scientific name of the kingdom in which the dwc: Taxon is classified.
phylum (Occurrence Extension)	The full scientific name of the phylum or division in which the dwc: Taxon is classified.
class (Occurrence Extension)	The full scientific name of the class in which the dwc: Taxon is classified.
order (Occurrence Extension)	The full scientific name of the order in which the dwc: Taxon is classified.
family (Occurrence Extension)	The full scientific name of the family in which the dwc: Taxon is classified.
genus (Occurrence Extension)	The full scientific name of the genus in which the dwc: Taxon is classified.
taxonRank (Occurrence Extension)	The taxonomic rank of the most specific name in the dwc: scientificName.
